# Role of an additional third screw in the fixation of transverse patellar fracture with two parallel cannulated screw and anterior wire

**DOI:** 10.1186/s12891-020-03744-x

**Published:** 2020-11-14

**Authors:** Chih-Wei Chang, Chih-Hsien Chen, Chun-Ting Li, Yen-Nien Chen, Tai-Hua Yang, Chia-Jung Chang, Chih-Han Chang

**Affiliations:** 1 Department of Orthopedics, National Cheng Kung University Hospital, College of Medicine, National Cheng Kung University, Tainan City, Taiwan; 2 Department of Orthopedics, College of Medicine, National Cheng Kung University, Tainan City, Taiwan; 3 Department of Orthopaedic Surgery, Tainan Municipal Hospital (Managed by Show Chwan Medical Care Corporation), Tainan City, Taiwan; 4 Institute of Geriatric Welfare Technology & Science, Mackay Medical College, New Taipei City, Taiwan; 5 Department of Physical Therapy, Asia University, 500, Lioufeng Rd, Wufeng, Taichung City, 41354 Taiwan; 6 Department of BioMedical Engineering, National Cheng Kung University, No.1, University Road, Tainan City, 701 Taiwan; 7 Skeleton Materials and Bio-compatibility Core Lab, Research Center of Clinical Medicine, National Cheng Kung University Hospital, College of Medicine, National Cheng Kung University, Tainan City, Taiwan; 8 Medical Device Innovation Center, National Cheng Kung University, Tainan City, Taiwan

**Keywords:** An additional third screw, Anterior wire, Cannulated screw, Finite element method, Patellar fracture

## Abstract

**Background:**

Two parallel cannulated screws along with an anterior wire to construct a tension band is a popular approach in transverse patellar fractures. However, the optimal screw proximity, either deep or superficial screw placements, remains controversial. Hence, a new concept of the addition of a third screw to form a triangular configuration along with the original two parallel screws was proposed in this study. Therefore, the biomechanical effect of the additional third screw on the stability of the fractured patella was investigated with finite element (FE) simulation.

**Methods:**

An FE knee model including the distal femur, proximal tibia, and fractured patella (type AT/OTA 34-C) was developed in this study. Four different screw configurations, including two parallel cannulated screws with superficial (5-mm proximity) and deep (10-mm proximity) placements and two parallel superficial screws plus a third deep screw, and two parallel deep screws plus a third superficial screw, with or without the anterior wire, were considered for the simulation.

**Results:**

Results indicated that the addition of a third screw increased stability by reducing the dorsal gap opening when two parallel screws were deeply placed, particularly on the fractured patella without an anterior wire. However, the third screw was of little value when two parallel screws were superficially placed. In the existence of two deep parallel screws and the anterior wire, the third screw reduced the gap opening by 23.5% (from 1.15 mm to 0.88 mm) and 53.6% (from 1.21 mm to 0.61 mm) in knee flexion 45° and full extension, respectively. Furthermore, in the absence of the anterior wire, the third screw reduced the gap opening by 73.5% (from 2 mm to 0.53 mm) and 72.2% (from 1.33 mm to 0.37 mm) in knee flexion 45° and full extension, respectively.

**Conclusion:**

Based on the results, a third cannulated screw superficially placed (5-mm proximity) is recommended to increase stability and maintain contact of the fractured patella, fixed with two parallel cannulated screws deeply placed (10-mm proximity), particularly when an anterior wire was not used. Furthermore, the third screw deeply placed is not recommended in a fractured patella with two parallel superficial screws.

## Background

Patellar fracture is a common scenario in orthopaedic clinics, and it usually occurs due to a sudden impact to the patella, such as a fall or traffic accidents [1, 2]. The major function of the patella is to transfer the quadriceps’ force to the tibia to generate knee motions. When a patellar fracture occurs, the function of force transfer is lost, and the knee joint becomes incapable of normal motions including flexion and extension. In serious cases, surgical intervention is recommended to maintain geometry, fix the fractured patella, and restore the flexion/extension mechanism of the knee joint for early rehabilitation exercise [3–5].

To date, many surgical approaches, such as tension band, modified tension band, cannulated screws with anterior wire, and even locking plate, have been developed for the fixation of patellar fractures [6–12]. Among these approaches, the approach of using two parallel cannulated screws with anterior wire has become popular because of favourable clinical outcomes [13–15]. Studies have indicated that the use of cannulated screw with anterior wire was superior to the modified tension and resulted in better clinical outcomes [11, 16]. However, the proximity of cannulated screws in the fixation of the fractured patella remains controversial. According to our previous studies [17, 18], the superficial placement of screw (5 mm depth from the anterior surface of the patella) was better than its deep placement (10 mm depth from the anterior surface of the patella) in reducing the fracture gap opening particular in knee flexion, whereas the deeply placed screw was better than the superficially located screw in maintaining the contact of the fracture site in knee full extension. Furthermore, in clinical practice, some surgeons prefer deep placement of screws to prevent bone rupture during drilling and insertion of screws, whereas others prefer superficial screw placement to obtain higher stability in the fixation of the patellar fracture with cannulated screws.

To strike a balance between superficial and deep screw proximities, a new concept of the addition of a third screw to form a triangular configuration along with the original two parallel screws was proposed in this study. Therefore, the first aim of this study was to investigate the biomechanical effect of the additional third screw on the fractured patella in different knee positions by using the finite element (FE) method. The second aim was to compare the mechanical differences of the fractured patella between the use and nonuse of the anterior wire with the cannulated screws. FE analysis is a numerical method for obtaining an approximate solution in engineering problems, and this method has been used in many orthopaedic studies [19, 20].

## Methods

Firstly, a knee model with a fracture patella fixed with the traditional fixation approaches, namely two parallel cannulated screws and the anterior wire, was developed and validated. After the validation succeeded, the proposed triangular screw configuration and anterior wire were applied on the fractured patella model and then analysed.

### Solid model

An intact knee model including the distal femur, proximal tibia, and patella was developed based on the computed tomography images of the open source from the US National Library of Medicine’s Visible Human Project. Images were taken of the knee full extension with a 1-mm interval, and bony contours in each image were retrieved by the higher gray value of the bone than that of the soft tissue in the Avizo software, Version 6.0 (VSG SAS, Bordeaux, France). The 3D model was developed by the lamination of obtained contours. Then, the 3D model was imported into a CAD software Solidworks 2019 (Dassault Systemes SolidWorks Corp., Waltham, MA, USA) to modify the knee position to flexion 45° and create the fracture pattern of the patella and screw implantations. A transverse fracture at the middle of the patella was created according to the type AT/OTA 34-C. Because the type is one of the indications of cannulated screw and tension band technique. The fracture was created with a virtual plane, and no gap existed between fragments. Thereafter, parallel cannulated screws with an anterior wire were used to construct a tension band to stabilize the fractured patella.

**Fig. 1:**
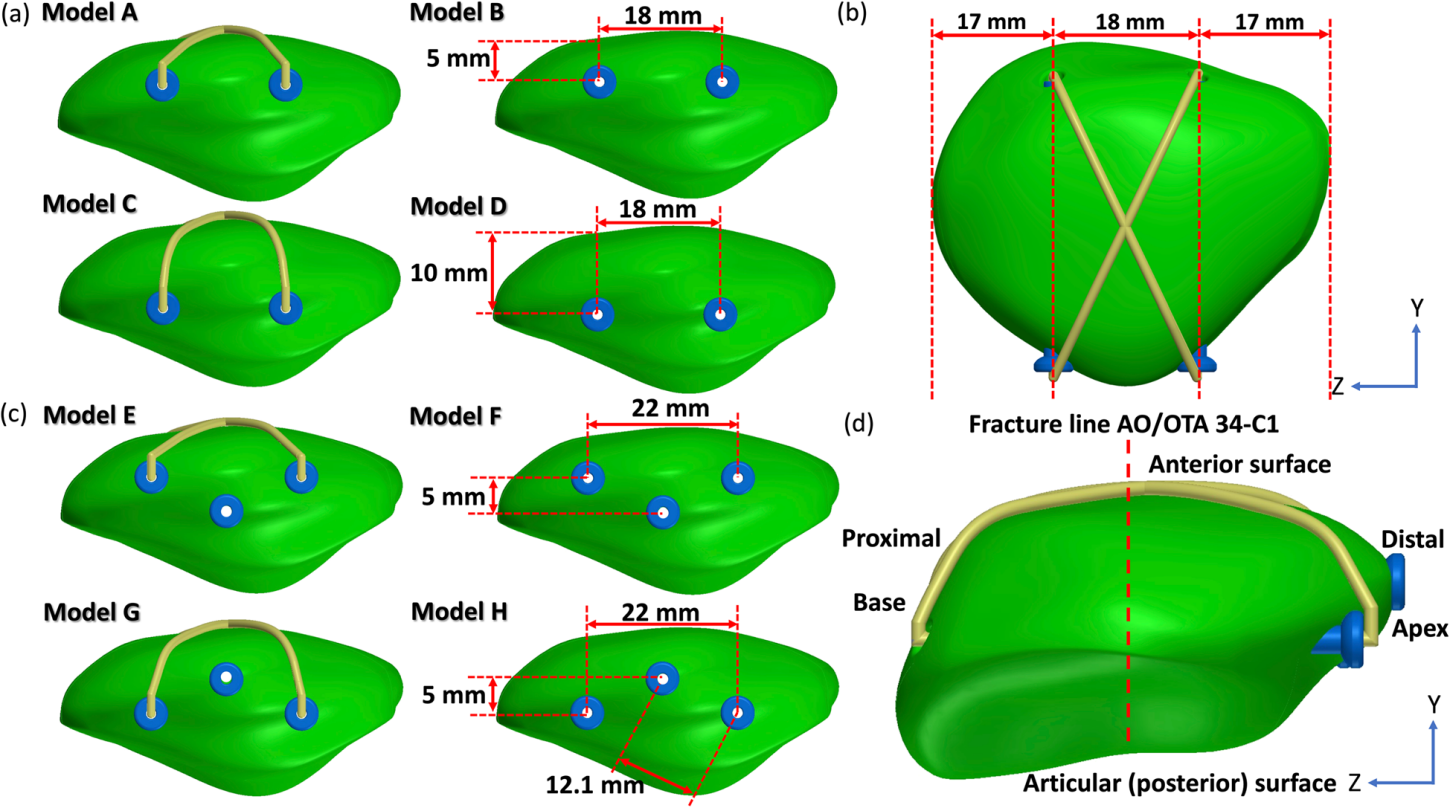
The fractured patella with two cannulated screws and anterior wire (**a** and **b**). The schematic of location of the two parallel deep screws, plus a superficial screw and two parallel superficial screws, plus a deep screw (**c** and **d**)

At first, different traditional approaches including two parallel superficial screws (at 5-mm depth from the anterior surface of the patella) at an 18-mm interval with (Model A) and without (Model B) the anterior wire, and two parallel deep screws (at 10-mm depth from the anterior surface of the patella) at an 18-mm interval with (Model C) and without (Model D) the anterior wire were used for validation (Fig. 1 a and b). The proximity of the two parallel cannulated screws was set based on the previous study [17, 21]. The interval of the two parallel screws was set to 18 mm (about one-third of the patella width) based on the previous study, too. The screws were created directly in the CAD software based on the currently used screws (207.642, DePuy Synthes, PA, USA) in clinical. The diameter of screws was uniformly set at 4 mm. The screw length was controlled with the tip under the cortical bone of the proximal patella. The thread length did not cross the fracture. Furthermore, an anterior wire of diameter 1.25 mm (Synthes Inc., West Chester, PA, USA) was created on the anterior surface of the fractured patella in a figure of eight. The wire was perfectly fit the anterior surface of the patella.

**Fig. 2:**
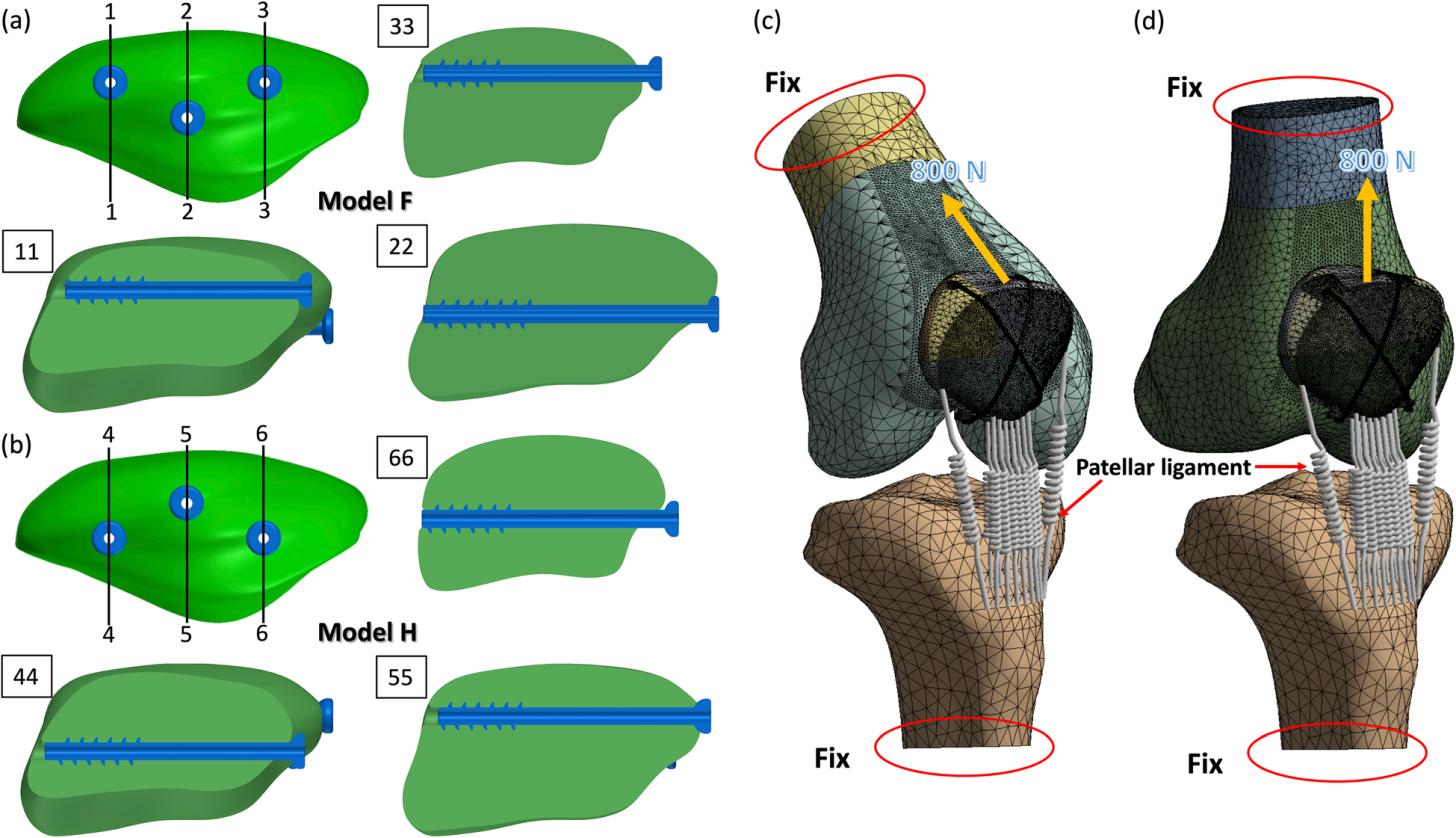
Sectional view of the model with the additional screw (**a** and **b**). Boundary conditions of the knee (**c**) at flexion 45° and (**d**) full extension

After the validation succeeded, the proposed triangular configuration by adding one more screw to the original two parallel screws was applied on the validated model. When the additional third screw was used, in order to increase the space between the screws, the interval between the original two parallel screws was increased from 18 to 22 mm. Therefore, another four models were developed, including two parallel superficial screws at a 22-mm interval plus a third deep screw in the middle of the two parallel screws with (Model E) and without (Model F) the anterior wire, and two parallel deep screws at a 22-mm interval plus a third superficial screw in the middle of the two parallel screws with (Model G) and without (Model H) the anterior wire (Figs. 1 c and d, and 2 a and b). Finally, eight different models with two parallel and triangular screw configurations with and without the anterior were used to evaluate the biomechanical effect of the additional third screw on the stability of the fractured patella.

### Finite element model

The models were imported into ANSYS Workbench 2019 R3 (Swanson Analysis, Houston, PA, USA) for mesh and simulation. The maximum edge lengths of the element for the wire, screw, patellar bone, patellar cartilage, and distal femur and the proximal tibia were set to 0.25 mm, 0.5 mm, 1 mm, 2 mm, and 5 mm, respectively, with the command “Sizing” in ANSYS. The contact areas of the patella with the wire and the screw and the distal femur with the patella were also locally refined with the commend “Sizing,” and the maximum length of the element edge was set to 0.5 mm and 1 mm, respectively. Overall, 519,421 nodes and 322,223 elements were used to mesh the model with two screws and the anterior wire. The other models were meshed using an identical setting.

The stiffness of the patellar ligament was about 2826 N/mm in the literature [22]. In the present study, the patellar ligament was constructed with 16 tension only springs, which connected the apex of the patella with the tibia tubercle (Fig. 2 c and d). Hence, the stiffness of each spring was uniformly set to 176.6 N/mm. The contact behaviours between the bone and bone (the fracture site), bone and metal (wire and patella and screw and patella), and metal and metal (wire and screw) were set to frictional surface to surface contact behaviours (contact 174 and target 170 in ANSYS) with frictional coefficients of 0.45, 0.3, and 0.2, respectively [23]. The contact behaviour between the patella and the distal femur condyle was set to frictionless. While the contact at the cross of the wire itself at the center of the anterior surface of the patella was not considered.

The material properties of the bone and cartilage were defined based on the literature (Table 1) [24–27]. The material properties of the stainless screw and wire were defined according to the engineering database of ANSYS. Bilinear hardening material property was used for the stainless in the present simulation. Hence, in the linear phase, the elastic modulus and Poisson ratio were set to 200,000 MPa and 0.3, respectively. The yield stress and tangent modulus were set to 250 MPa and 1450 MPa, respectively.

**Table 1:** The material properties used in this study

	Elastic modulus (MPa)	Poisson Ratio	Reference
Femur	19,000	0.3	[24]
Tibia	17,000	0.3	[25]
Patella			
Cortical bone	1000	0.3	[26]
Trabecular bone	207	0.3	[26]
Cartilage	50	0.1	[27]

### Boundary condition

For validation, a 400 N force (parallel to the long axis of the femur) was applied on the base of the fractured patella with the anterior wire (Models A and C) in knee flexion 45°. The bilateral ends of the knee were totally fixed. The displacement of the loading site on the proximal fragment under 400 N was compared with that in the Bryant’s study [28]. In addition, a ramp load parallel to the long axis of the femur, was applied to the base of the fractured patella without the anterior wire (Models B and D) until 2 mm displacement at the loading site both in knee full extension and flexion 45°. The bilateral ends of the knee were totally fixed, too. The loading 2 mm displacement and the linear stiffness (the ratio of the applied load to the maximum displacement of the loading site) were compared with that of Dargel’s result [29].

For the comparison of the biomechanical effect between the traditional and the proposed approaches, two different degrees of force, namely 800 N and 400 N, were used in the simulation. The 800 N tension force parallel to the long axis of the femur was applied to the base of the fractured patella with the anterior wire (Models A, C, E and G), in knee flexion 45° (Fig. 2c) and full extension (Fig. 2d) to simulate the quadriceps force during normal walking [30]. A force of 400 N in the same direction was applied to the fractured model without the anterior wire (Models B, D, F and H) [29], because the fractured patella at 10-mm screw proximity and without the anterior wire was unable to bear an 800 N loading in knee flexion 45°. The applied force was in the same direction with the long axis of the femur, hence the Q angle was considered in the simulation.

The maximum displacement of the fragment and the maximum gap opening distance were used as indices to assess the stability of the fractured patella with various screw configurations. The contact area maintained between the fragments was used to judge the ability of screw configurations to stabilize the fractured patella. The fractured patellar fragments were initially in contact with each other without any gap and separated from each other after addition of the load. Greater the contact area maintained after the static balance, better was the stability achieved by the screws.

## Results

### Validation

The linear stiffness and load with 2-mm deformation of the present FE model with 10-mm screws placement without the anterior wire were similar to that in Dargel’s study, whereas the results with 5-mm screw proximity were larger than that in Dargel’s study (Table 2). Furthermore, results of the present FE model with the anterior wire and Bryant’s results were at the same level under 400 N force in flexion 45°. The results indicated that our model was able to represent the responses of the fractured patella with cannulated screws and an anterior wire.

**Table 2:** Comparison of the stiffness and load with 2 mm displacement in the experiment and FE calculations

With anterior wire			
	Bryant	Model A (5-mm screw placement with the wire)	Model C (10-mm screw placement with the wire)
Flexion 45° Displacement (mm) under 400 N	1.2 ± 0.34	1.63	1.75
Without anterior wire			
	Dargel	Model B (5-mm screw placement without the wire)	Model D (10-mm screw placement without the wire)
Full extension			
Linear stiffness (N/mm)	240.67 ± 28.44	253.44	243.31
Load with 2 mm displacement (N)	549.64 ± 56.18	703.1	504
Flexion 45°			
Linear stiffness (N/mm)	147 ± 50.96	266.88	170.81
Load with 2 mm displacement (N)	351.35 ± 119.88	758.9	340

### Fragment displacement and gap opening distance

**Fig. 3:**
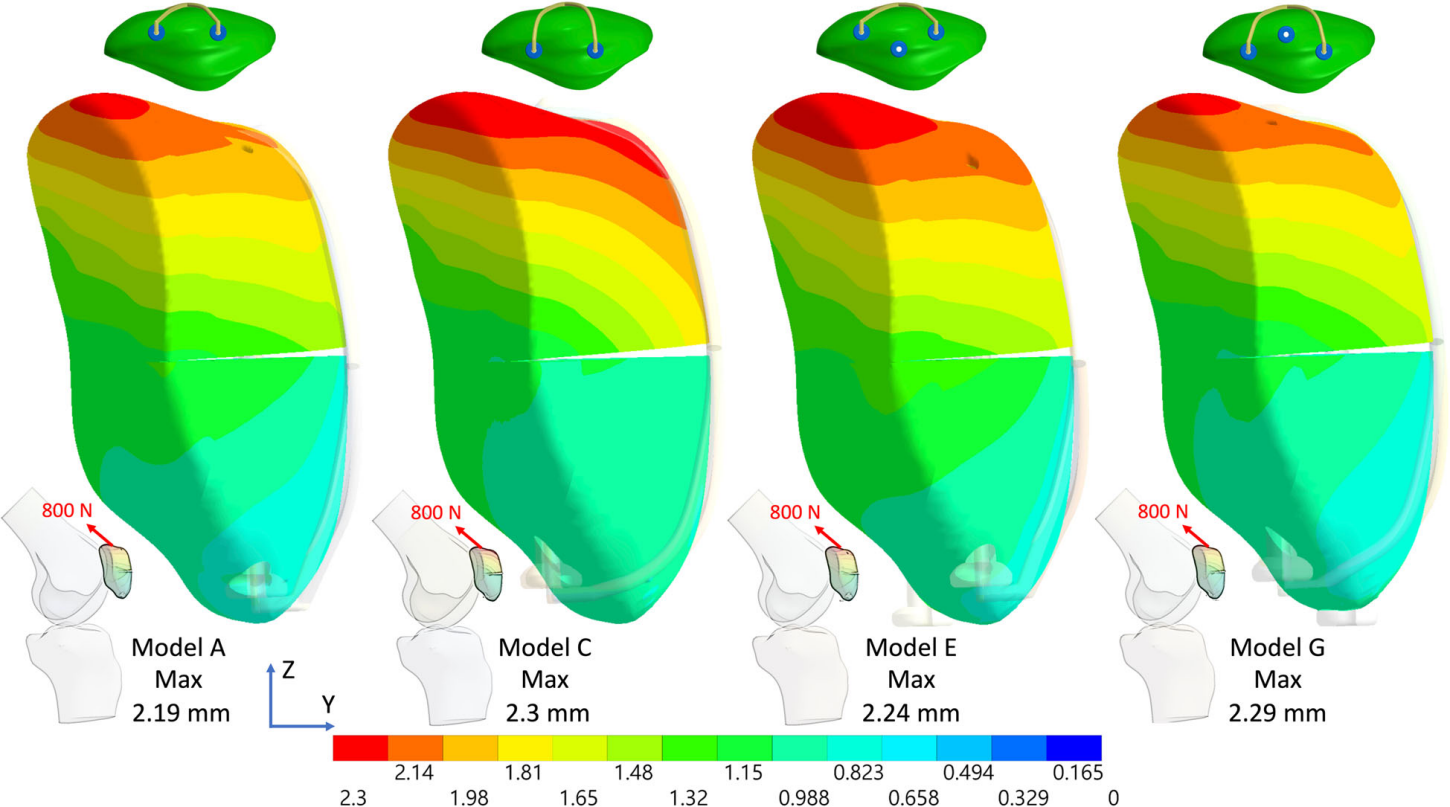
Total displacement of the fractured patella with deep and superficial screws with the anterior wire and the third screw in knee flexion 45° under an 800 N force. (Lateral view)

**Fig. 4:**
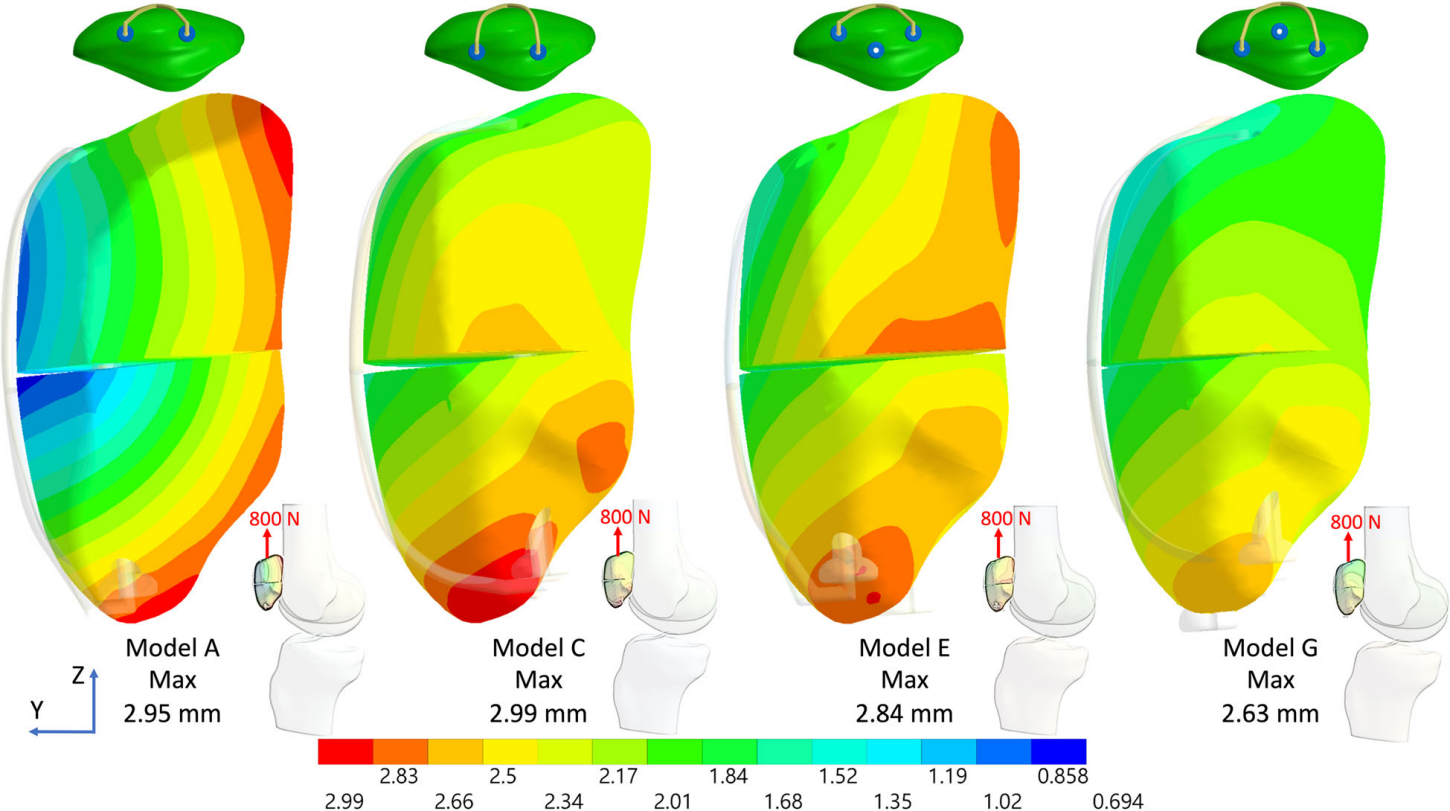
Total displacement of the fractured patella with deep and superficial screws with the anterior wire plus the third screw in knee full extension under an 800 N force. (Medial view)

**Fig. 5:**
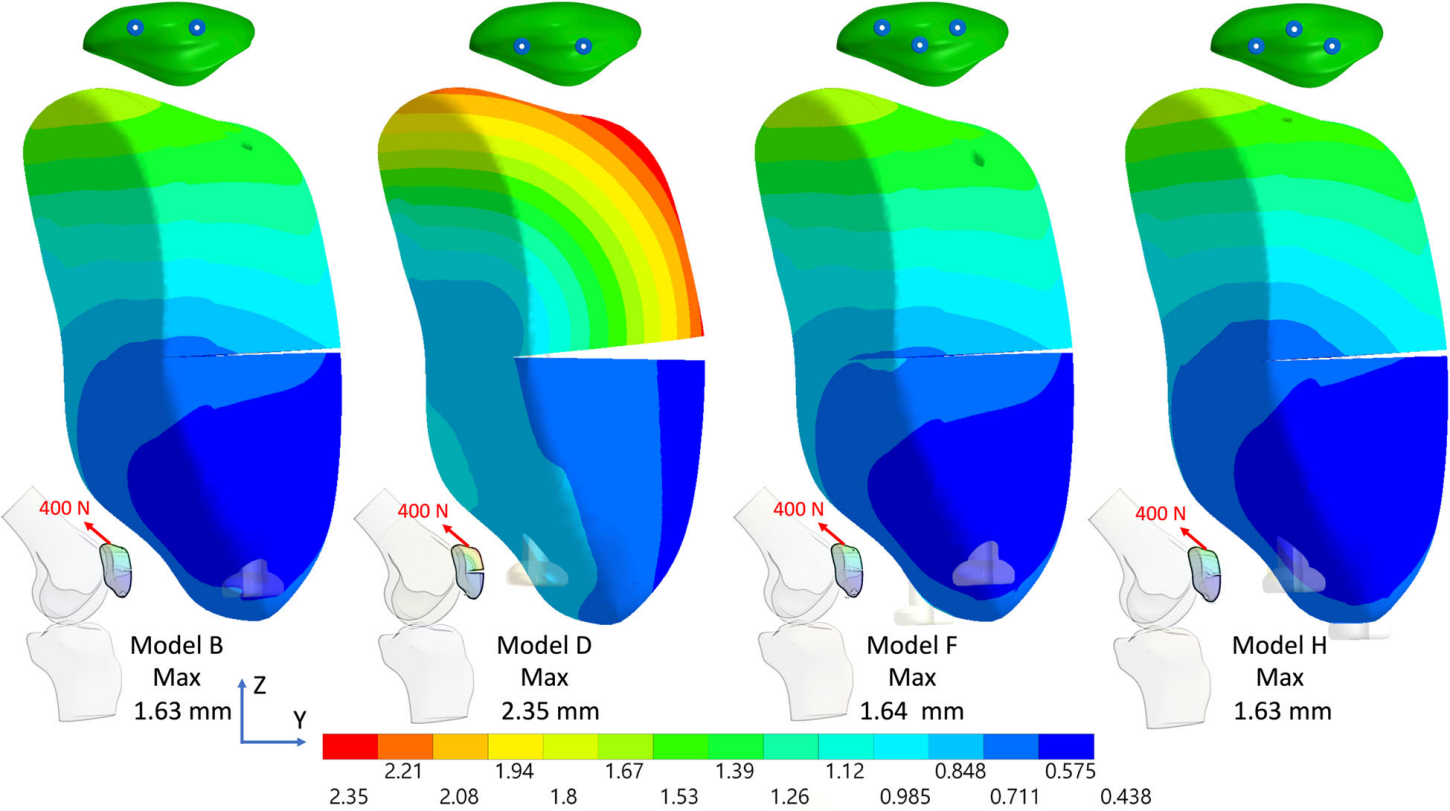
Total displacement of the fractured patella with deep and superficial screws without the anterior wire plus the third screw in knee flexion 45° under a 400 N force. (Lateral view)

**Fig. 6:**
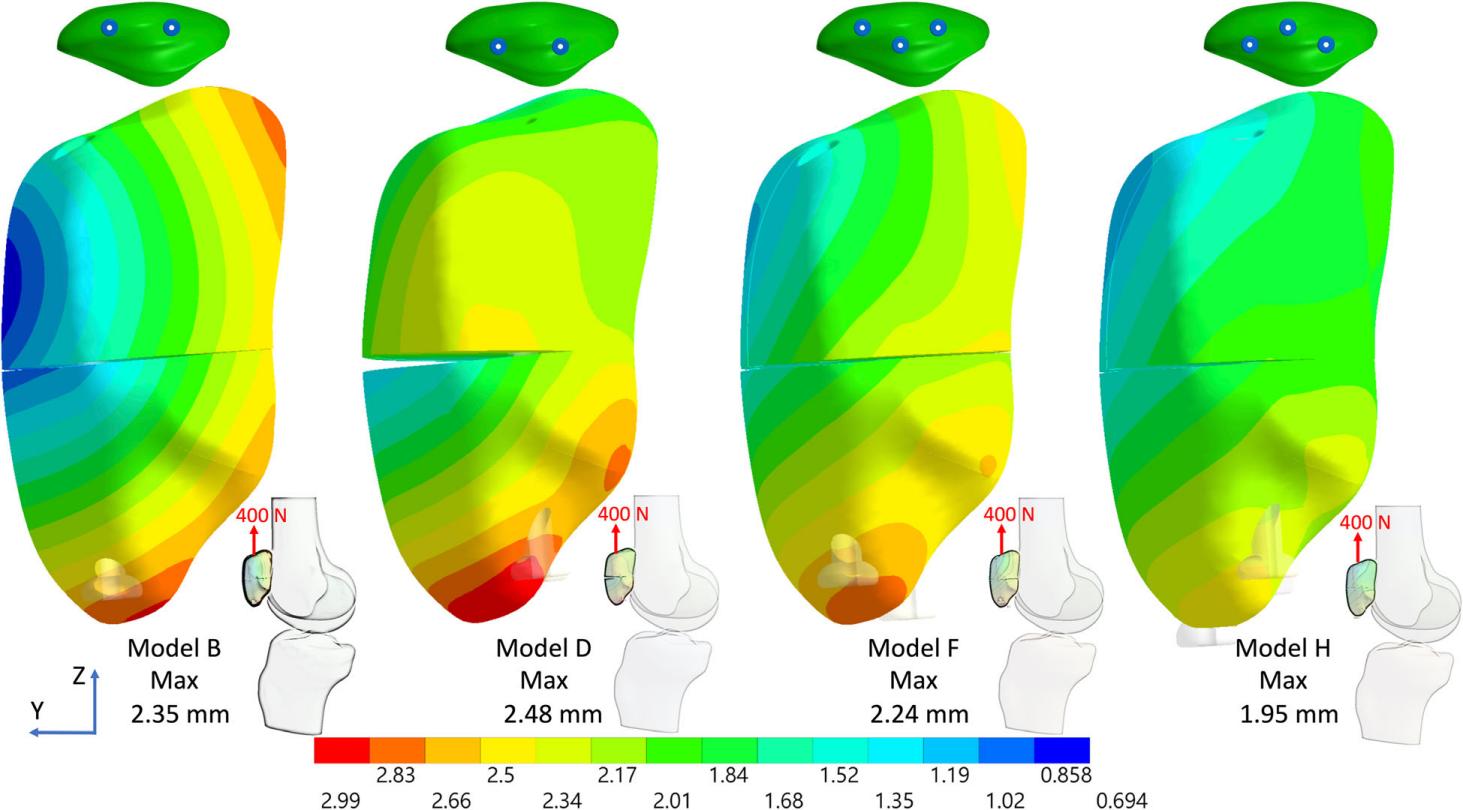
Total displacement of the fractured patella with deep and superficial screws without the anterior wire plus the third screw in knee full extension under a 400 N force. (Medial view)

**Fig. 7:**
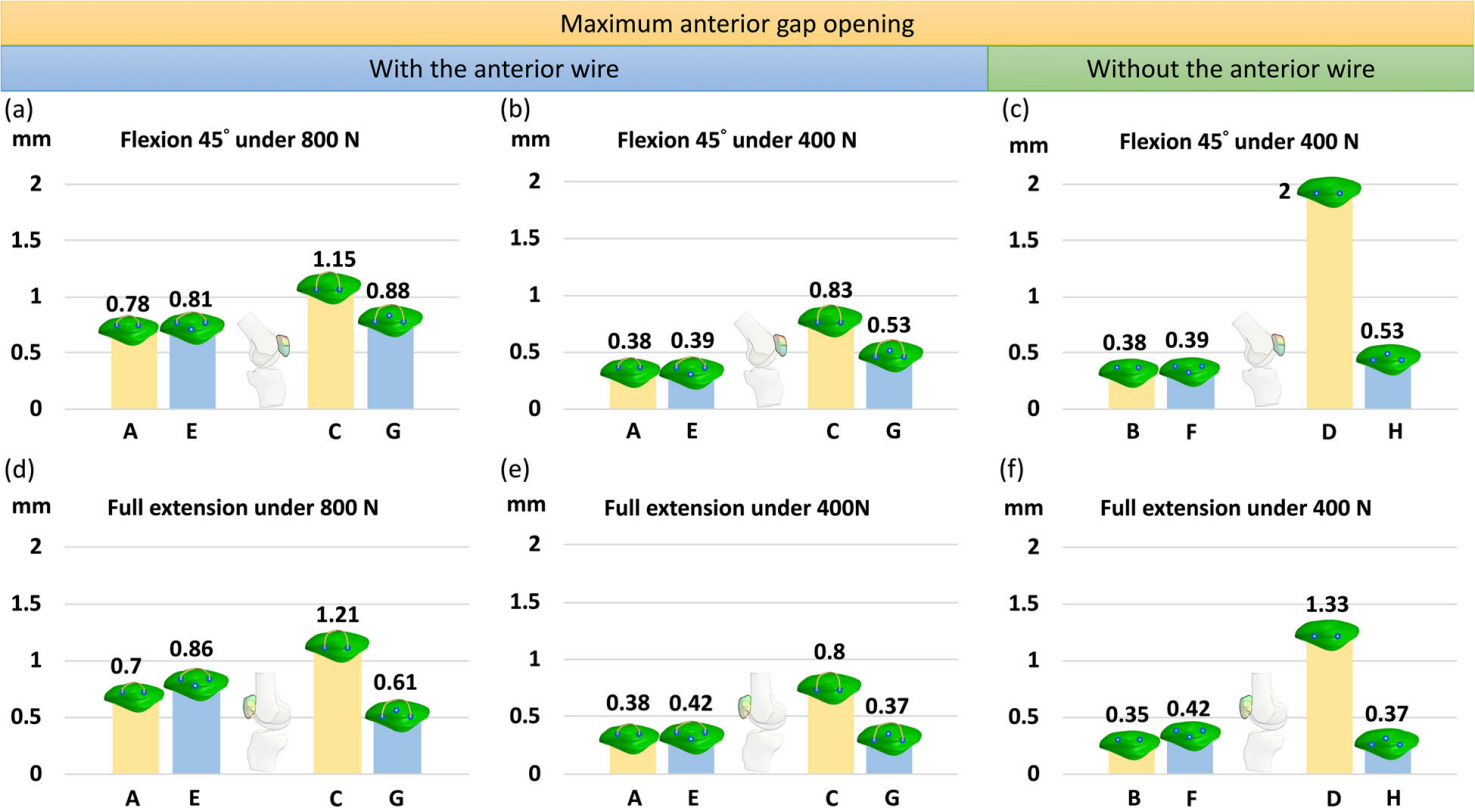
Gap opening distance in knee flexion (**a**, **b**, and **c**) and extension (**d**, **e**, and **f**) under 800 N (left column) and 400 N (middle column) with the anterior wire, and 400 N without the anterior wire (right column)

The results indicated that the additional third screw increased stability by reducing the fragment displacement and the anterior gap opening when the original two parallel screws were deeply placed (Figs. 3 and 4), particularly when the anterior wire was not used (Figs. 5 and 6). Furthermore, after using the additional third screw, the stability of the two parallel deep screws was increased to almost the same as that with two parallel superficial screws. However, the third screw was of little value for the fractured patella with two parallel screws superficially placed either with or without the anterior wire. When the original two parallel screws were deeply placed, the third screw reduced the gap opening by 23.5% (Fig. 7a), 36.1% (Fig. 7b), and 73.5% (Fig. 7c), respectively under 800 N and 400 N force with wire and 400 N without wire in knee flexion 45°. Additionally, in the knee full extension, the third screw reduced the gap opening by 49.6% (Fig. 7d), 53.8% (Fig. 7e), and 72.2% (Fig. 7f), respectively, under 800 N and 400 N with the wire and under 400 N without wire. The maximum differences of gap opening between the triangular configuration without the wire and two parallel superficial screws plus wire was just 0.15 mm (from 0.38 to 0.53) under identical 400 N.

### Contact area

**Fig. 8:**
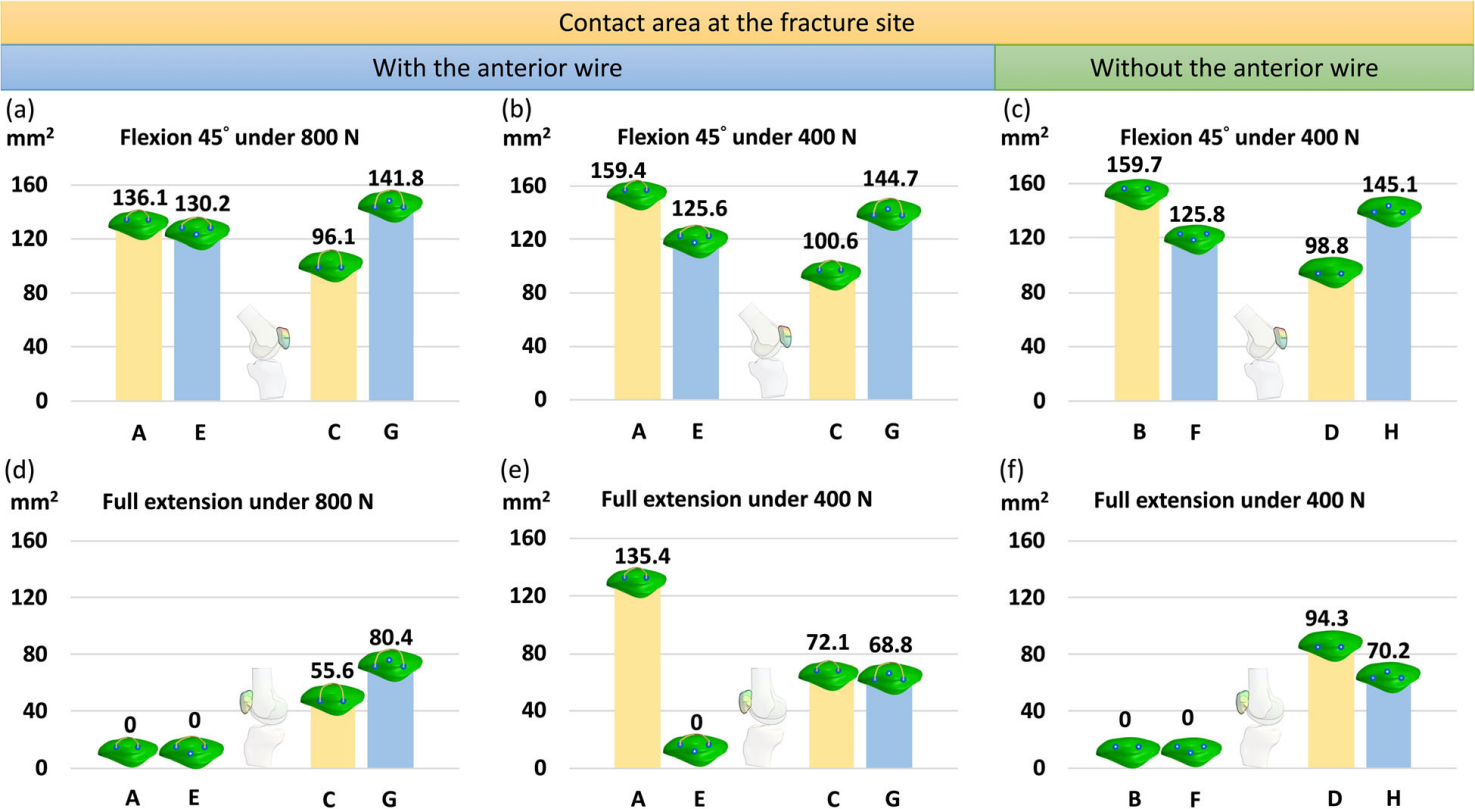
Contact area between the fragments in knee flexion (**a**, **b**, and **c**) and extension (**d**, **e**, and **f**) under 800 N (left column) and 400 N (middle column) with the anterior wire and 400 N without the anterior wire (right column). Zero contact area meant that the two fragments are completely separated

The additional third screw also maintained the contact area between the patellar fragments with deep screws in knee flexion 45° (Fig. 8a–c), whereas, in the knee full extension, the third screw was still able to partially maintain the contact area between the patellar fragments with deep screws (Fig. 8d–f). The patellar fragments were completely separated after the use of the third screw in two parallel superficial screws. The contact area was increased by 47.6% (from 96.1 mm^2^ to 141.8 mm^2^) after using the additional third screw in two parallel deep screws and wire in flexion 45°under 800 N.

## Discussion

The optimal fixation approach for transverse patellar fracture is still unknown, because the mechanical responses of the patella are complicated during knee motions. Each of the existing approaches have advantages and disadvantages [11, 31, 32]. Hence, a new concept of application of an additional third screw to construct a triangular configuration with the traditional two parallel cannulated screws was proposed in the present study to improve the weakness of either the superficial or deep screw placements. This approach was able to improve the disadvantage while maintaining the strength of the original single screw proximity. This approach is a combination of superficial and deep screw proximities and provides an additional choice, instead of the original either superficial or deep proximity, for the surgeon in the management of transverse patellar fracture with cannulated screws. In this approach, the surgeon can now overcome the challenge of selecting single screw proximity for the surgical fixation of transverse patellar fractures.

The effect of the additional third screw on increasing the stability was especially obvious in the two parallel deep screws, particularly without the anterior wire, because the added third screw increased the ability to resist torsional loading on the fractured patella. In two parallel deep screws, the loadings on the patella finally developed a torsion load after static balance both in knee flexion 45° and full extension. The superficial (leading) surface is on the tension side, whereas the articular surface is on the compression side. Therefore, no screw was in place to resist torsion when screws were deeply placed without the anterior wire [17, 18]. Therefore, adding a screw on the tension side was helpful for the two deeply placed screws to resist the torsional loading and thereby reduce the gap opening distance. Alternatively, when the original two parallel screws and the anterior wire were superficially placed on the tension side in knee flexion 45°, the additional third screw on the compression side was helpless to increase the resistance to torsional loading. Otherwise, in two parallel superficial screws, the applied load developed a tensile load on the fractured patella in full extension and the patellar fragment sliding along the screws and separated from each other [17, 18]. In such cases, the additional third screw was also not useful in preventing the separation while maintaining the contact between the fragments.

Nevertheless, using the additional third screw with two parallel deep screws could maintain the contact between proximal and distal fragments, both in knee full extension and flexion 45°, because the loading finally developed a torsional load in the original deep screw placement. Initially, the patellar fragments should be in contact with each other without any gap after fracture reduction, and the contact area should decrease with increasing applied load. A proper fixation should be able to maintain the contact of the fractured patella to promote direct bone healing (also called primary bone healing) of the fracture [33, 34]. Direct bone healing requires an anatomical reduction of the fracture site, keep the contact of the fragment without any gap as much as possible with a stable fixation. Then the direct remodelling of lamellar bone, the Haversian canals and blood vessels achieves the direct bone healing after the fixation. The direct healing is often the primary goal in the management of open reduction and internal fixation surgery. In the present study, although the value of the contact area with the two parallel superficial screws plus a third screw was similar to that with two parallel deep screws plus a third screw, two parallel superficial screws plus a third screw were unable to prevent the patellar fragments from separating completely during full extension. Therefore, the additional third screw was not suggested in case of two parallel superficial screws.

The role of the additional third screw is similar to that of the anterior wire when the original two parallel screws were deeply placed, because they were all on the same side. Although the location of the anterior wire is more superficial than the third screw, the diameter of the third screw (4 mm) was much larger than the anterior wire (1.25 mm). The increased outer diameter increased the ability to resist the external force, particularly to the torsional load. From a mechanical view, moment of inertia of a cylinder is proportional to the third power of the outer diameter. Therefore, increase the outer diameter is very helpful to increase the stiffness and resistance ability to the loading. Hence, the effect of the additional third screw was more obvious than that of the anterior wire in the present simulation, in spite of shorter distance from the articular surface than the wire. Hence, the additional third screw reduced the gap opening but increased the contact area more than the anterior wire in the fractured patella with two deep parallel screws under identical (400 N) force.

In addition to the improved stability, the use of an additional third screw has another advantage –the feasibility of minimally invasive surgery over the wire. The insertion of cannulated screw can be executed via minimally invasive techniques, whereas the wiring of the encircling of the wire often required open manners [35]. Furthermore, the additional screw can be implanted with less iatrogenic soft tissue trauma compared to anterior wiring. Less surgical damage should be considered as the first priority to fasten the recovery of the patients. In our previous study [18], the deep cannulated screw alone was not suggested in the fixation of patellar fracture because of its weakness to the torsional load. Hence, the anterior wire was suggested for deep screw placement, and thus an anterior incision of the soft tissue above the patella was almost inevitable. In such situation, the proposed additional third screw is strongly suggested to substitute the anterior wire and forms a triangular screw configuration with the original two parallel deeply placed cannulated screws. Because the contribution of the additional third screw was more than that of the anterior wire in the current simulation but less surgical damage.

The aim of the surgical fixation of the patellar fracture is to construct a rigid fixation for early rehabilitation soon after the surgical intervention [3, 36]. Although the present triangular fixation formed with two deep and one superficial screw placement has more advantages than both the superficial or deep screw placement, this approach is relatively feeble in maintaining the contact of the fractured patella in knee extension without the anterior wire. Hence, the quadriceps setting exercise or isometric quadriceps contraction exercise in full knee extension should be avoided. Additionally, the disadvantages of using the additional third screw including the challenge of placing three screws in the limited space of the patella and the loosening of the screws in the absence of the wire. The surgeon must risk bony breakage and wound while applying the presented strategy using three cannulated screws to patients with a relatively small or osteopenic patella. Therefore, the proposed approach of three cannulated screws is not suggested for a relatively small or osteopenic patella. However, due to the promising biomechanics in the present study, the suitable size of the patella for three parallel cannulated screws in a triangular configuration is worth consideration in future studies.

This study has some unavoidable limitations. First, the distal femur and proximal tibia were fixed at different knee flexion degrees, and the meniscus and the articulation of the distal femur and the proximal tibia were not considered in the simulation. Furthermore, the alignment of the knee joint and patella in flexion 45° was manually adjusted and was not based on the images in flexion. Second, in the present simulation, only a worst-case scenario was considered and the force magnitudes in flexion and extension were uniform. The alerting of the force and moment at the beginning and mid-stage of knee flexion were ignored. Third, the material properties of the cortical and cancellous bones were simplified as linear elastic, isotropic, and homogeneous. The trabecular architecture was not modelled. Finally, the manipulated tension of the wire was not considered.

## Conclusion

The effect of implementing an additional third screw was highly related to the original screw proximity in the fixation of patellar fractures with cannulated screws. Based on the present results, a third cannulated screw superficially placed (5 mm from the anterior surface of the patella) with two parallel cannulated screws, deeply placed (10 mm from the anterior surface of the patella) is recommended in the management of patellar fracture when the anterior wire was not used. While a third screw deeply placed is not recommended for addition to the fractured patella along with two superficial parallel cannulated screws whether the anterior wire was used or not.

## Data Availability

The data used during the present study are available from the corresponding author on reasonable request.

## Abbreviation

FE: Finite element
